# Within-Plant Variation in *Rosmarinus officinalis* L. Terpenes and Phenols and Their Antimicrobial Activity against the Rosemary Phytopathogens *Alternaria alternata* and *Pseudomonas viridiflava*

**DOI:** 10.3390/molecules26113425

**Published:** 2021-06-05

**Authors:** Maria Bellumori, Marzia Innocenti, Federica Congiu, Gabriele Cencetti, Aida Raio, Felicia Menicucci, Nadia Mulinacci, Marco Michelozzi

**Affiliations:** 1 Department of Neurofarba, Division of Pharmaceutical and Nutraceutical Sciences, University of Florence, Via U. Schiff 6, Sesto Fiorentino, 50019 Florence, Italy; maria.bellumori@unifi.it (M.B.); marzia.innocenti@unifi.it (M.I.); federica.congiu@tiscali.it (F.C.); 2 Institute of Biosciences and Bioresources, National Research Council of Italy (CNR), Via Madonna del Piano 10, Sesto Fiorentino, 50019 Florence, Italy; gabriele.cencetti@ibbr.cnr.it (G.C.); marco.michelozzi@cnr.it (M.M.); 3 Institute for Sustainable Plant Protection, National Research Council of Italy (CNR), Via Madonna del Piano 10, Sesto Fiorentino, 50019 Florence, Italy; aida.raio@ipsp.cnr.it; 4 Institute for the Chemistry of OrganoMetallic Compounds, National Research Council of Italy (CNR), Via Madonna del Piano 10, Sesto Fiorentino, 50019 Florence, Italy; felicia.menicucci@iccom.cnr.it

**Keywords:** rosemary extracts, enantiomeric monoterpenes, young and mature tissues, bacteria, fungi, bioactive compounds, rosmarinic acid, GC/MS, HPLC/DAD/MS, natural products

## Abstract

This study investigated within-plant variability of the main bioactive compounds in rosemary (*Rosmarinus officinalis* L.). Volatile terpenes, including the enantiomeric distribution of monoterpenes, and phenols were analyzed in young and mature foliar, cortical and xylem tissues. In addition, antimicrobial activity of rosmarinic acid and selected terpenes was evaluated against two rosemary pathogens, *Alternaria alternata* and *Pseudomonas viridiflava*. Data showed that total concentration and relative contents of terpenes changed in relation to tissue source and age. Their highest total concentration was observed in the young leaves, followed by mature leaves, cortical and xylem tissues. Rosmarinic acid and carnosic acid contents did not show significant differences between leaf tissues of different ages, while young and mature samples showed variations in the content of four flavonoids. These results are useful for a more targeted harvesting of rosemary plants, in order to produce high-quality essential oils and phenolic extracts. Microbial tests showed that several terpenes and rosmarinic acid significantly inhibited the growth of typical rosemary pathogens. Overall, results on antimicrobial activity suggest the potential application of these natural compounds as biochemical markers in breeding programs aimed to select new chemotypes less susceptible to pathogen attacks, and as eco-friendly chemical alternatives to synthetic pesticides.

## 1. Introduction

The Mediterranean basin is endowed with a wealth of aromatic plants, including rosemary (*Rosmarinus officinalis* L.), an evergreen shrub whose health benefits have been recognized since ancient times. There is a rising awareness of consumers toward health foods for disease prevention, and rosemary is considered one of the most interesting sources of phytochemicals that could provide health benefits [1]. This aromatic plant is added directly to food as fresh leaves or in the form of concentrated extracts. Two groups of molecules are mainly responsible for the biological activities and beneficial health properties of the rosemary plant: the volatile terpenes as main constituents of essential oils, and the phenolic compounds [2]. Essential oil, as well as phenolic extracts of rosemary, are recognized as safe by the FDA [3] and EFSA [4].

Essential oils are a complex mixture of volatile and semi-volatile compounds, including monoterpenes, that can represent up to 90% of the oil, and sesquiterpenes as the main components [5]. Other minor constituents are esters, ketones, small phenols and alcohols [6,7]. A number of studies report antioxidant, anti-inflammatory and antimicrobial properties of *R. officinalis* essential oil [8,9,10]. Furthermore, the strong influence of terpenes on the sensorial quality and shelf-life of several foods has been widely recognized [2].

Rosemary is used not only to obtain essential oils but also to produce phenolic leaf extracts containing a pool of compounds responsible for several biological properties, as demonstrated by in vitro experiments and in vivo animal tests [11]. Three main groups of phenolic compounds characterize these extracts: rosmarinic acid, a group of minor flavonoids and phenolic diterpenes such as carnosic acid and carnosol. The main bioactivities related to these phenolic compounds are anti-inflammatory [12,13], chemo-preventive [14,15] and anti-proliferative [16]. Recently, protective effects towards neurological disorders have been noted for rosmarinic and carnosic acids [17], as well as an ability to decrease the risks related to obesity, diabetes and metabolic syndrome [18,19].

Jordan et al. [20] explained that the highest antimicrobial and antioxidant activities of rosemary essential oils and phenolic extracts collected at the fruit maturation phase, compared to full bloom, were due to variations in the contents of several terpenes and phenols. In general, variations occurring in rosemary terpene and phenol phenotypes are the product of the interaction of the genes with the environment and are also due to plant ontogeny and phenology [21,22].

Although changes in constitutive or induced plant secondary metabolites are known to play a major role in innumerable defensive functions against insects, herbivores and plant pathogens, the number and structural diversity of these defensive chemicals provide a myriad of significant ecological roles [23,24].

Chemo-systematic studies showed that the total absolute amounts of monoterpenes of essential oils and resins can be influenced by abiotic factors, while the relative percentages of constitutive monoterpenes in mature tissues are under strong genetic control and scarcely affected by abiotic stresses [23,25]. Therefore, monoterpenes have often been used as biochemical markers to characterize plant species, hybrids, provenances, families and clones [26].

These variations in chemical composition of essential oils and phenols are important to characterize superior chemotypes for potential industrial applications in food, pharmaceutical, perfumery and cosmetic fields.

Although monoterpenes are widely studied as the main constituents of essential oils, based on our knowledge, there are very few studies on enantiomeric monoterpenes of rosemary. Particularly, two and four works on chiral monoterpenes respectively in foliar tissues [27,28] and in essential oils obtained from leaves or aerial parts of the plant [29,30,31,32] were reported in the literature. It should be pointed out that this is the first study showing enantiomeric variations of rosemary monoterpenes in different tissues of different age, and these variations are known to play different ecological roles and induce different sensorial perceptions [24].

The aims of this study were to investigate on the changes in content of bioactive compounds of rosemary plant and to evaluate their activity against some typical rosemary pathogens. Specific objectives were: (i) to determine variation in total concentrations and relative contents of terpenes and phenols among different types of tissue (foliar, cortical and xylem), (ii) to investigate the effects of tissue age on the content of terpenes and phenols, (iii) to determine enantiomeric terpene distribution within-plant and (iv) to evaluate the antimicrobial activity of selected terpenic compounds (including some chiral monoterpenes) and rosmarinic acid, against the fungus *Alternaria alternata* and the bacterium *Pseudomonas viridiflava*. These pathogens are known to induce heavy damages on aerial parts of the rosemary plants, causing important economic losses in nursery [33,34]. A better knowledge on terpenes and phenolic content in the different tissues can be useful for a more targeted harvesting of rosemary plants, in order to produce high-quality essential oils and phenolic extracts. At the same time, knowledge of antimicrobial properties of rosemary tissue components can be usefully applied for breeding programs aimed to select less susceptible chemotypes to pest and diseases and to produce eco-friendly formulates, alternatives to synthetic pesticides, less harmful to human and environmental health.

## 2. Results

### 2.1. Terpenes

**Figure 1:**
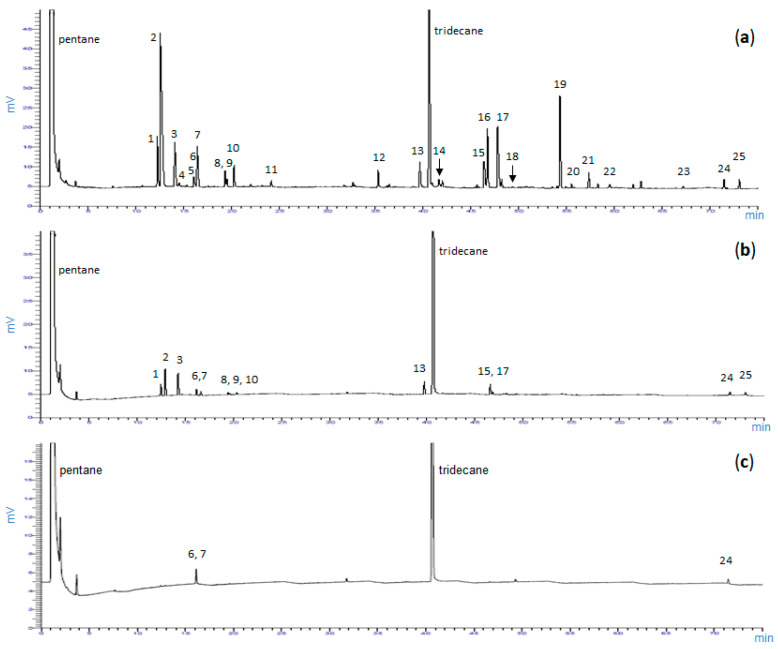
Representative GC-MS chromatogram of foliar (**a**), cortical (**b**) and xylem (**c**) tissues of *Rosmarinus officinalis* L. showing chiral and non-chiral volatile terpenoids. 1, (−)-α-pinene; 2, (+)-α-pinene; 3, camphene; 4, sabinene; 5, myrcene; 6, (+)-β-pinene; 7, (−)-β-pinene; 8, (−)-limonene; 9, (+)-limonene; 10, p-cymene; 11, 1,8-cineole; 12, (−)-linalool; 13, camphor; 14, terpinen-4-ol; 15, borneol; 16, (−)-bornylacetate; 17, (−)-verbenone; 18, geraniol; 19, β-caryophyllene; 20, geranyl acetate; 21, α-humulene; 22, eugenol; 23, thymol; 24, carvacrol; 25, caryophyllene oxide. Tridecane as internal standard. Metabolite identification obtained by both mass spectrum and retention time index.

Figure 1 compares three representative GC chromatographic terpene profiles of foliar (a), cortical (b) and xylem (c) tissues, and shows enantiomeric monoterpenes, namely (+)/(−)-α-pinene, (+)/(−)-β-pinene, (+)/(−)-limonene, (−)-linalool, (−)-bornylacetate and (−)-verbenone. All the 25 terpenes identified are also reported in Table S1 of the Supplementary Materials.

The enantiomers (+)-camphene and (+)-linalool were detected in trace amounts in a few samples and, therefore, were not considered in the elaboration of data. The two forms of β-pinene were distinguished only in a few samples of foliar tissue, where they occurred in considerable amounts. Some difficulties were encountered to obtain a complete chiral chromatographic separation of cortical and xylem tissue samples, mainly because only trace amounts of each single enantiomer were detected.

#### 2.1.1. Total Concentrations of Terpenes (TCT)

**Figure 2:**
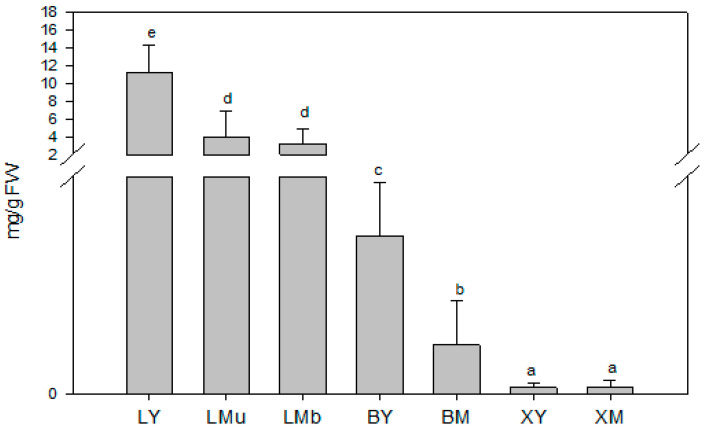
Total contents of volatile terpenoids (mg/g fresh weight (FW)) in young and mature tissues and different tissue sources from *Rosmarinus officinalis* L. Data are means of 9 replicates + SE (standard error). Different letters indicate significant differences (by the Mann–Whitney U-test, *p* < 0.05). L, leaf; B, cortex; X, xylem tissue; Y, young tissue; M, mature tissue. Samples collected from the upper part (u) and at the base (b) of the branch are indicated respectively, as “u” and “b”.

As shown in Figure 2, significant variations in TCT were detected among samples collected from different plant tissues and of different ages (N = 63, *χ*^2^ = 54.8, df = 6, *p* < 0.001). The greatest amounts of TCT occurred in young leaves (LY), mature leaves of the upper part of the branch (LMu) and mature leaves of the base of the branch (LMb), while the lowest were observed in the xylem tissue samples (XY, young xylem tissue; XM, mature xylem tissue). Regarding tissue age, TCT was up to three times higher in young leaves (LY) than in mature leaves (LMu and LMb samples).

#### 2.1.2. Relative Contents of Terpenes (Terpene Profiles)

**Figure 3:**
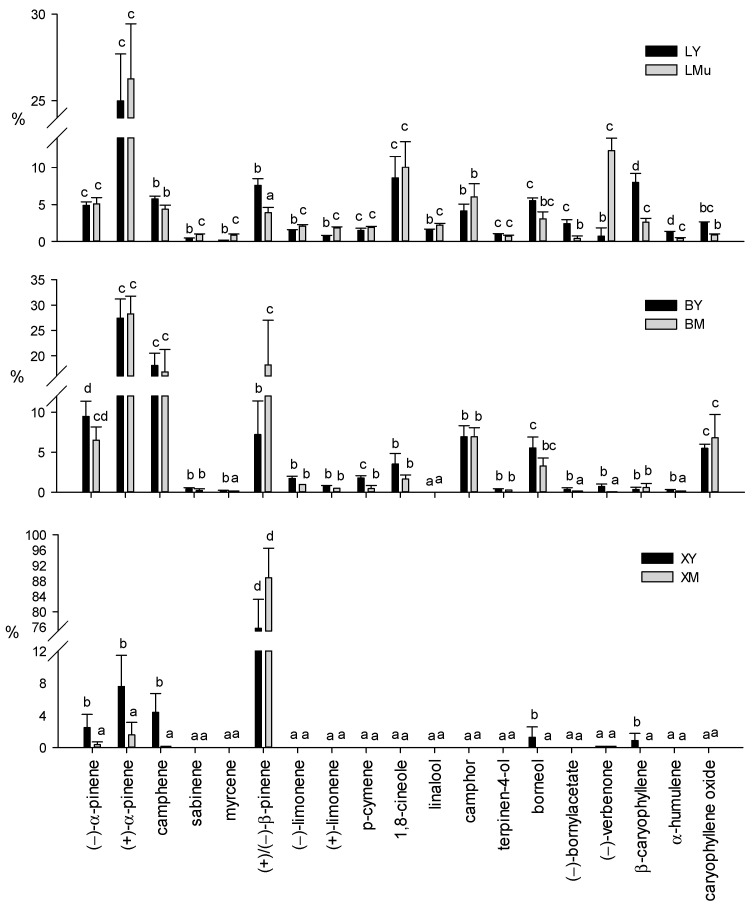
Relative contents of volatile terpenoids in young and mature tissues and different tissue sources from *Rosmarinus officinalis* L. Data are means of 9 replicates + SE (standard error). Different letters indicate significant differences (by the Mann–Whitney U-test, *p* < 0.05) in the relative content of each single terpene between young and mature samples of different tissue sources. L, leaf; B, cortex; X, xylem tissue; Y, young; M, mature. Samples collected from the upper part of the branch are indicated with “u”.

The terpene profiles of different tissue sources and ages are shown in Figure 3. No significant differences in the relative content of each compound were detected between mature leaves collected from two different parts of the branch (LMu and LMb samples)—only the LMu graph is shown in the figure.

Differently from what was observed within mature leaves, the Kruskal–Wallis ANOVA test showed significant differences in the relative contents of all the compounds within samples of different source and different tissue age (Supplementary Material, Table S1).

The relative contents of all the volatile terpenes showed significant differences among different tissue sources. In particular, as shown in Figure 3, (+)-α-pinene was the most abundant compound in all leaf samples (LY: 25.0%; LMu: 26.3%; LMb: 26.54%) and in the cortex (BY: 27.4%; BM: 28.3%), while (+)/(−)-β-pinene showed the highest relative contents in xylem tissues (XY: 75.7%; XM: 88.8%). In mature leaves, the relative contents of (+)-β-pinene in LMu (3.6%) and LMb (2.7%) were significantly higher than (−)-β-pinene (LMu 0.4%; LMb 0.7%). On the contrary, the percentage of (−)-β-pinene in young leaves (LY) was significantly higher (6.0%) than (+)-β-pinene (1.6%) (data not shown). Other abundant constituents of leaves were (−)-verbenone (LMu: 12.27%; LMb: 13.1%), 1,8-cineole (LY: 8.6%; LMu: 10.0%; LMb: 8.7%) and β-caryophyllene (LY: 7.9%). Differently, (−)-camphene (BY: 18.1%; BM: 16.8%), (+)/(−)-β-pinene (BM, 18.2%) and (−)-α-pinene (BY, 9.5%) were the major compounds in cortical tissue samples.

The young xylem tissue (XY) samples showed a limited number of terpenes, including (+)/(−)-β-pinene (over 75%), (+)-α-pinene (7.6%) and (−)-camphene (4.4%), and minor constituents such as (−)-α-pinene (2.5%), borneol (1.3%) and β-caryophyllene (0.9%). In XM tissue, in addition to (+)/(−)-β-pinene (close to 90%), only two other monoterpenes, (+)-α-pinene (1.6%) and (−)-α-pinene (0.4%), were detected.

Other minor terpenes (α-phellandrene, α-terpinene, γ-terpinene, terpinolene, α-terpineol, myrtenol, geraniol, geranyl acetate, α-humulene, eugenol, thymol and carvacrol) identified in very small amounts (less than 0.5%) were omitted from Figure 3.

From our findings, it emerged that the different ages significantly modified the terpene composition of the foliar tissue (Supplementary Table S1). The highest contents of (+)/(−)-β-pinene, (−)-bornylacetate, β-caryophyllene and α-humulene were detected in young leaf (LY) samples, also characterized by significantly lower percentages of sabinene, myrcene, (−)-limonene, (+)-limonene, (−)-linalool and (−)-verbenone than mature leaves. On the contrary, mature leaves (LMu and LMb) were significantly richer in (−)-verbenone (Figure 3).

In addition, significant differences in the relative contents of myrcene, (+)/(−)-β-pinene, p-cymene, (−)-bornylacetate, (−)-verbenone and α-humulene were observed between the two cortex samples of different ages (Figure 3). All these terpenes showed significantly higher concentrations in the young cortical tissue samples than the mature ones, with the only exception being (+)/(−)-β-pinene.

Among the few terpenes detected in xylem tissue, the XY samples showed significantly higher contents of (−)-α-pinene, (+)-α-pinene, (−)-camphene, borneol and β-caryophyllene than the XM samples (Figure 3). As already observed for cortical tissues, the amount of (+)/(−)-β-pinene did not show significant differences between samples of the two ages.

### 2.2. Phenolic Compounds

The content of phenolic compounds was investigated in the foliar, cortical and xylem tissues of rosemary. The HPLC profile of the extracts from cortical and xylem tissues showed a lack of those phenolic compounds typically present in rosemary leaves (e.g., rosmarinic acid, carnosic acid, carnosol, etc.), while only negligible amounts of caffeic acid derivatives and flavonoids were detected. Consequently, Table 1 shows only the quantitative distribution of phenols in young and mature leaves (LY and LMu).

**Figure 4:**
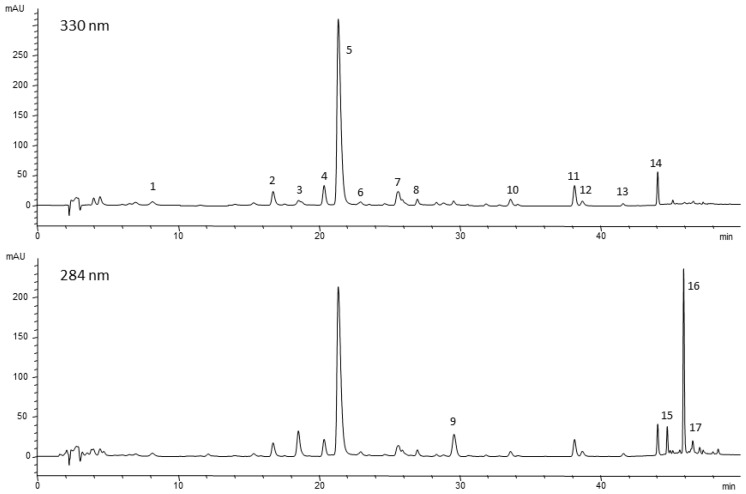
HPLC profiles at 330 and 284 nm of the ethanol extract from fresh young foliar tissue (LY). 1, caffeic acid; 2, isoorientin (luteolin 6-*C*-glucoside); 3, hesperidin; 4, homoplantaginin (hispidulin 7-*O*-glucoside); 5, rosmarinic acid; 6, luteolin 7-*O*-glucuronide; 7, isoscutellarein 7-O-glucoside; 8, flavonoid 1; 9, rosmanol derivative; 10, dihydroxy-dimethoxyflavone; 11, cirsimaritin; 12, flavonoid 2; 13, genkwanin; 14, 4′-methoxytectochrysin; 15, carnosol; 16, carnosic acid; 17, methyl carnosic acid.

The applied multistep elution method separated seventeen different compounds (Figure 4 and Table S1 of the Supplementary Materials). Among them, rosmarinic acid, several minor flavonoids such as aglycones (cirsimaritin and genkwanin) and glycosides (hispidulin 7-*O*-glucoside and isoscutellarein 7-*O*-glucoside), and the diterpenoidic constituents, mainly carnosic acid and its oxidation products, were identified.

The main phenols of this plant, rosmarinic acid and carnosic acid and its derivatives, did not show significant differences between the leaf tissues of the two different ages. As shown in Table 1, the Mann–Whitney U-Test showed significant differences (N = 18, df = 1) between LY and LMu samples in the content of four flavonoids: isoorientin (*χ*^2^ = 7.8), homoplantaginin (hispidulin 7-*O*-glucoside) (*χ*^2^ = 12.8), cirsimaritin (*χ*^2^ = 5.5) and genkwanin (*χ*^2^ = 9.8). Overall, these results did not permit to detect possible markers linked to the age of rosemary foliar tissue among the phenolic compounds.

### 2.3. Effect of Selected Terpenes and Rosmarinic Acid on A. alternata and P. viridiflava

**Figure 5:**
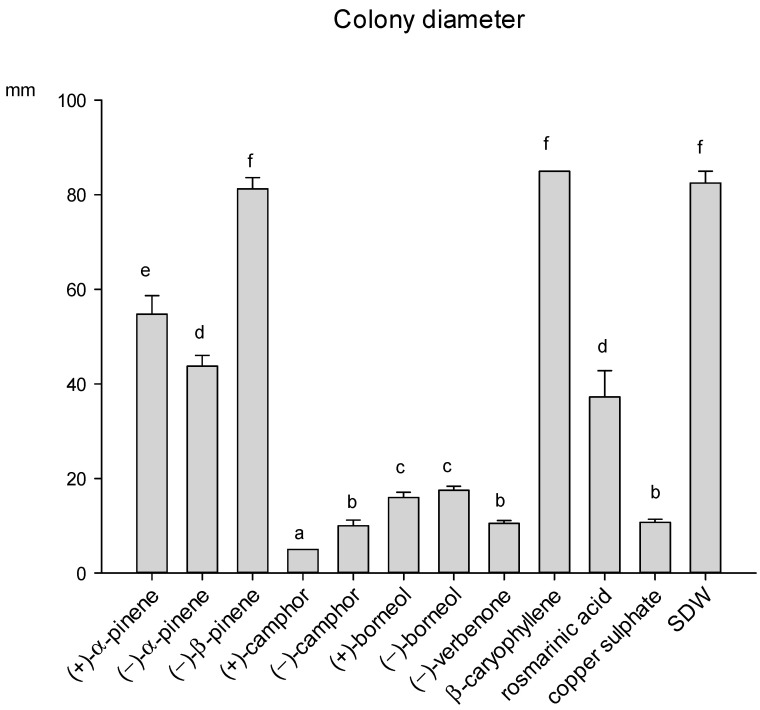
Growth of *A. alternata* colony diameter (mm) in the presence of different compounds of rosemary leaves, after 14 days of incubation at 25 °C. Data are means of 6 replicates + SE (standard error). Different letters indicate significant differences (by the Mann–Whitney U-test, *p* < 0.05) among the tested compounds.

The growth of the phytopathogenic fungus *A. alternata* was affected by eight out of the ten compounds tested, since β-caryophyllene and (−)-β-pinene were totally ineffective. (+)-camphor was the most effective compound, showing an inhibition activity higher than copper sulphate, while the antifungal activity of (−)-camphor and (−)-verbenone was instead comparable to that of the fungicide. (+)/(−)-borneol also determined a strong inhibition of the fungal growth (Figure 5). Rosmarinic acid, the only phenolic compound tested, showed a partial inhibiting activity against *A. alternata.* The fungus produced a diffusible dark-brown pigment in response to the presence of rosmarinic acid in the medium, as shown in Figure S1 of the Supplementary Materials.

**Figure 6:**
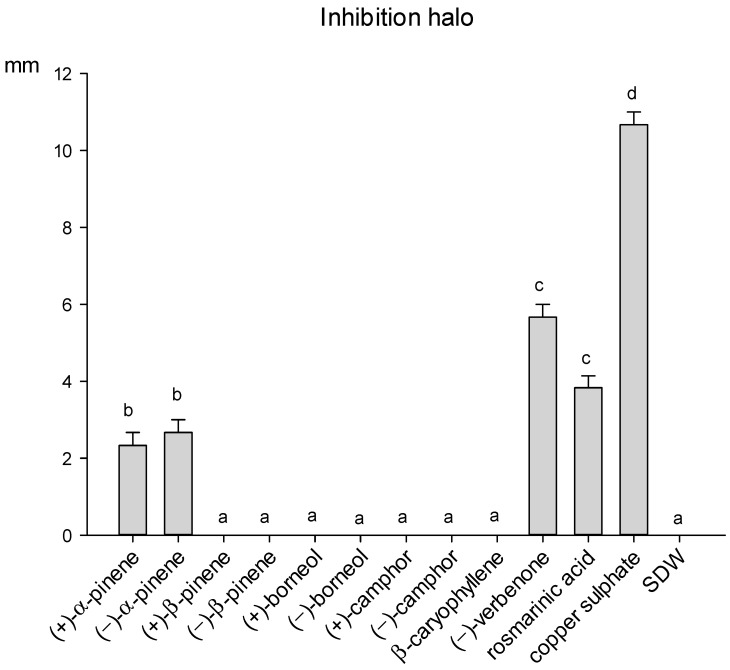
Size of inhibition halo (mm) determined by different compounds of rosemary leaves against the bacterium *P. viridiflava*, after 48 h of incubation at 27 °C. Data are means of 6 replicates + SE (standard error). Different letters indicate significant differences (by the Mann–Whitney U-test, *p* < 0.05) among the tested compounds.

Only α-pinene enantiomers, (−)-verbenone and rosmarinic acid inhibited the growth of the bacterium *P. viridiflava*, while the remaining seven compounds resulted totally ineffective (Figure 6). Verbenone showed the highest antibacterial activity, producing an inhibition halo of 5.6 mm. However, its inhibition activity was significantly lower than copper sulphate.

Again, rosmarinic acid induced the production of a dark-brown diffusible pigment in the medium inoculated with *P. viridiflava*, similar to that observed for *A. alternata* (Supplementary Material Figure S2). Both *A. alternata* and *P. viridiflava* colonies originated healthy colonies when transferred from the plates where the dark pigment was observed to new agar plates.

### 2.4. Determination of the Minimum Inhibitory Concentration (MIC)

Results regarding MIC determination by the microdilution method are reported in Table 2 and in Table S3 of the Supplementary Materials. MIC values determined by the agar well-diffusion method for the terpenic compounds that were not soluble in PDB medium used to grow the fungus are reported in Table 3.

*A. alternata*: The MIC for (+)-α-pinene and (−)-verbenone was 0.625%, while it was 0.313% for (−)-β-pinene and 1.25% for rosmarinic acid. Since the growth medium appeared opaque after the addition of rosmarinic acid, for this compound, the MIC was not determined by visual inspection but only by the spectrophotometer absorbance values, as reported in Table S3 of the Supplementary Materials. Moreover, at the end of the incubation, for rosmarinic acid at the concentration of 0.313%, the production of a dark pigment in the suspension was observed (Supplementary Materials Table S3), as shown by the increased value of absorbance. Methanol had no effect on the fungal growth. For the two camphor enantiomers and borneol, the MIC was determined by the agar well-diffusion assay and was 0.625% for both enantiomers and 1.25% for borneol (Table 3).

*P. viridiflava*: The MIC value for (+)-α-pinene, (−)-verbenone and rosmarinic acid was 1.25%. A partial growth reduction of the bacterium was detected by visual inspection for verbenone at the concentration of 0.625% (Supplementary Materials, Figure S3). For copper sulphate, the use of resazurin allowed to determine a MIC of 0.313%, while it was 0.625% by visual inspection. Methanol inhibited the bacterial growth only at the concentration of 5%, and the next dilutions did not affect the bacterial growth (Supplementary Materials, Figure S3).

## 3. Discussion

### 3.1. Terpenes

In general, our data showed that TCT and relative contents of terpenes changed in relation to different tissue sources and tissue ages.

The highest TCT was observed in the leaves, while the lowest levels were observed in the xylem tissue samples. Our results agree with the data of Yosr et al. [21], which revealed a significantly higher yield of essential oils in leaves than stems and flowers. TCT was much higher in young leaves than mature leaves, in agreement with data in the literature showing the highest contents in developing leaves [23,35]. Terpene biosynthesis per gram of leaves is a high energy-expensive process for the plant, that cannot maintain high levels of these defensive substances in all tissues or organs during the whole life cycle. Therefore, terpenes are preferentially accumulated in young tissues as they are generally more vulnerable to the attack of herbivores [23].

It is worth noting that rosemary essential oil production is usually obtained via steam distillation of small branches with young and mature leaves of the current season’s growth. Our data showed that leaf tissues represented over half (about 66%) of the total biomass of branches, followed by xylem tissues (about 24%) and cortical tissues (about 10%). Therefore, leaves representing the major source of biomass with the highest TCT are the organs that contribute to a greater extent to the chemical profile of essential oil and its final quality.

No differences in relative terpene contents were detected between mature leaves collected from two different parts of the branch. These results are in agreement with previous data reporting that relative contents of constitutive monoterpenes in mature tissues remain stable and can be used as biochemical markers in chemotaxonomic studies [21,36].

On the contrary, differences in the relative terpene contents were found between samples of different tissue sources and different tissue age. Such variations, that are defined as epigenetic variations, are reported in the scientific literature [23,36].

Differences in terpene profiles were observed among the three tissue sources: terpene profiles of the leaves were characterized by high relative contents of (+)-α-pinene and 1,8-cineole, and high relative contents of (−)-verbenone and β-caryophyllene were also detected in mature and young leaves, respectively. Cortical terpene profiles showed high relative contents of (+)/(−)-α-pinene, camphene, (+)/(−)-β-pinene, camphor and caryophyllene oxide in mature bark, while xylem tissue was characterized by the presence of (+)/(−)-β-pinene as the major compound, reaching up to almost 89%. In other plant species, variations in terpene profiles were reported as those detected between cortical and foliar tissues of Mediterranean pine species of group “*halepensis*” (*Pinus halepensis* Mill., *Pinus brutia* Ten. and *Pinus eldarica* Medw.). In particular, limonene was present in higher relative content in cortical than foliar tissue of *P. eldarica*, and this monoterpene represented a powerful biochemical marker to distinguish this pine species from *P. halepensis* and *P. brutia* [37]. Cheng et al. [38] also reported differences in essential oil yield and terpene composition among four tissues (leaf, bark, heartwood and sapwood) of the Japanese cedar (*Cryptomeria japonica* D. Don). They also proposed that the sesquiterpenes δ-cadinene, isoledene and γ-muurolene might provide resistance to four wood decay fungi and three pathogenic fungi.

From our findings, it emerged that the age significantly modified the terpene composition of the different tissue sources.

Few reports regarding studies on the variations in terpene profiles between young and mature tissues are available, and most of these works are related to leaf tissue. In this study, it was shown that the highest contents of (+)/(−)-β-pinene, (−)-bornylacetate, β-caryophyllene and α-humulene were detected in young leaf samples, while mature leaves showed high relative content of (−)-verbenone (Figure 3).

Some of the changes in terpene contents related to the leaf age are consistent with the function of these molecules to protect the plant against the attack of pests and diseases, in fact, it has been hypothesized that changes were determined by herbivore selection pressures [23].

Young cortical tissue samples had higher relative contents of myrcene, p-cymene, (−)-bornylacetate, (−)-verbenone and α-humulene than the mature ones, with the only exception being (+)/(−)-β-pinene. As already mentioned in the results, only a few terpenes were detected in young tissue of xylem showing higher relative contents of (−)-α-pinene, (+)-α-pinene, (−)-camphene, borneol and β-caryophyllene than mature tissue samples.

Our data were in good agreement with the work by Yosr et al. [21], who observed that terpene composition of rosemary plants changed mainly between leaves and stems, and also among samples collected at different phenological stages.

According to the terpene profiles of leaves (Figure 3), the plants used in this study can be classified as an α-pinene chemotype of *Rosmarinus officinalis* L., known to be widely present in Italy and Morocco [6,39]. Previous reports indicate that verbenone has also been used to define the verbenone chemotype, and the rosemary essential oil called *verbenoniferum* is described as containing over 15% of this terpene [39]. Even in mature leaves, that showed the maximum concentration for this compound, (−)-verbenone always remained below 13.1% of the total volatile terpenes.

Variations in the enantiomeric composition of several monoterpenes occurred between various tissue sources at different developmental stages. It must be pointed out that until now, no information on enantiomeric distribution has been available for cortex and xylem tissues of rosemary. This current study shows that (+)-α-pinene was the predominant enantiomer, in agreement with data in the literature [27,31]. For the other monoterpenes, the enantiomeric distribution detected in our samples were partially in agreement with data in the literature. (−)-camphene and (−)-linalool were the predominant enantiomers, in agreement with data on fresh leaf samples reported by Larkov et al. [27]; on the contrary, Yassa and Williams [40] and Satyal et al. [31] found (+)-camphene and (+)-linalool to be dominant. In our data, no enantiomeric excess was observed for limonene, in agreement with that reported by Satyal et al. [31], while this monoterpene was enantiomerically pure, 100% (+)-limonene and 100% (−)-limonene, as described in the studies by Tomi et al. [30] and Yassa and Williams [40], respectively.

The different enantiomers of chiral monoterpenes can exhibit diverse ecological roles [24] and different sensory attributes. Furthermore, the enantiomer composition of monoterpenes can be used as a biochemical marker of authenticity of rosemary aroma, for quality assessment of the essential oils and to identify the origin of the oil.

### 3.2. Phenolic Compounds

The content of phenolic compounds was reported only for leaf samples, since extracts from cortical and xylem tissues showed traces or negligible amounts of caffeic acid derivatives and flavonoids. The main phenols of this plant, rosmarinic acid and carnosic acid and its derivatives, were not significantly different between the leaf tissues of the two different ages. Young and mature leaf samples showed differences in the content of isoorientin, homoplantaginin (hispidulin 7-*O*-glucoside), cirsimaritin and genkwanin. Our data partially agree with the study by Yosr et al. [21], showing that polyphenols were mainly accumulated in leaves and flavonoids did not vary significantly during different growth stages of the leaves.

### 3.3. Antimicrobial Activity against A. alternata and P. viridiflava

Antimicrobial activity of rosemary essential oil and its components has been widely investigated against several pathogens of clinical origin, spreading mainly as food contaminants and responsible for dangerous diseases to humans, and some plant pathogens [41,42,43]. To our knowledge, however, the antimicrobial activity of rosemary essential oil compounds has not yet been evaluated against the fungus *A. alternata* and the bacterium *P. viridiflava*, two polyphagous plant pathogens able to infect rosemary plants [33,34]. The two species were tested for their sensitivity to rosmarinic acid and to the terpenic compounds identified in this study as the main components of rosemary essential oil. Except for β-caryophyllene and (−)-β-pinene, the remaining seven compounds showed a significative antifungal activity, that in the case of camphor and (−)-verbenone, inhibited the growth of *A. alternata* at the same rate as copper sulphate, a compound commonly used as a pesticide to control bacterial and fungal pathogens and allowed in organic farming [44]. On the other hand, the bacterium *P. viridiflava* was inhibited only by the two α-pinene enantiomers, (−)-verbenone and rosmarinic acid. However, their efficacy was significantly lower than copper sulphate. It is known that Gram-negative bacteria (including *Pseudomonas* species) show low sensitivity to the antimicrobial activity of terpenic compounds, since their outer membrane contains hydrophilic polysaccharides (LPS) which create a barrier towards macromolecules and hydrophobic compounds [45]. This could explain the negative response of *P. viridiflava* to most of the terpenes assayed in this study. Rosmarinic acid, that is a naturally occurring phenolic compound in a number of plants belonging to the *Lamiaceae* family, showed a certain effectiveness against both the plant pathogens tested in this study, in which it induced the production of a dark-brown diffusible pigment. Indeed, some microbial species are able to produce the dark pigment melanin through the activation of the enzyme laccase that catalyzes the oxidative polymerization of phenolic compounds. The activation of melanin synthesis is known as a cell survival mechanism for several micro-organisms [46]. Most probably, the dark-brown pigment secreted in the growth medium by *A. alternata* and *P. viridiflava* is a melanin-like compound produced by the two pathogens as a defense mechanism against rosmarinic acid. In fact, both pathogens were inhibited in the growth but not devitalized by rosmarinic acid. Since polyphenols are produced in plants to prevent microbial infections, it is conceivable that during host–pathogen co-evolution, a resistance mechanism (laccase synthesis) was transferred from the plant to the pathogen. This hypothesis is supported by phylogenetic studies that have evidenced that the fungal laccases are closely related to plant laccases [46].

Results obtained from the antimicrobial tests performed in this study highlight new possible strategies to control the two rosemary pathogens in the nursery: rosemary varieties at high content of (−)-verbenone and α-pinene should be less sensitive to the attack of the two pathogenic species considered in this study, then a selection on the basis of terpene profile could help to screen among the available commercial rosemary genotypes for resistance to pathogens. Copper-based formulates are widely used in conventional as well as in organic farming. However, repeated and excessive use of copper can lead to accumulation in soil, where it is toxic to many macro- and micro-organisms, while the accumulation in the food web may determine toxicity to humans [44]. Then, the use of copper in agriculture is quite controversial and the development of alternative formulates is strongly needed. Results regarding the antimicrobial activity of terpenic compounds obtained in this study are very promising, however further studies are needed for the development of terpene-based formulates. The development of new verbenone/α-pinene-based formulates may represent an alternative way to use the available chemical pesticides for controlling rosemary pathogens and most probably also other bacterial and fungal plant pathogens. The inclusion of terpenes in a solid matrix should extend the release time and enhance the water solubility of these highly volatile hydrophobic compounds [47].

## 4. Materials and Methods

### 4.1. Plant Material

The samples were collected from nine rosemary adult plants growing in a nursery located in Montevarchi (Italy). Foliar, cortical and xylem tissue samples were collected at determined heights and horizontal positions on the plants, following the sampling procedure indicated by Squillace [36]. Mechanically damaged or diseased plants were not sampled.

Samples of young, newly developing tissues from the plant parts of the current-year growth, and one-year-old mature tissues were collected separately and brought to the laboratory. The samples are indicated by letters as follows: L = leaf, B = cortex, X = xylem tissue, Y = young and M = mature tissues. Furthermore, mature leaves were collected in the upper part (u) and at the base (b) of the branches.

A total group of 63 samples of these tissues (LY, LMu, LMb, BY, BM, XY, XM) were submitted to the GC analysis, and 18 samples from leaves (LY and LMu) were selected for the quantitative evaluation of the phenols by HPLC/DAD/MS.

### 4.2. Extraction of Terpene Volatile Compounds

Fresh leaves, cortical and xylem tissues were frozen in liquid nitrogen and ground into a porous ceramic mortar (grinding time about 1 min). The grinding is made to allow the breakdown of cellular structures containing terpenic constituents, avoiding the loss of these substances, which are extremely volatile. For each sample, 0.5 g of fresh ground material was extracted with 3 mL of *n*-pentane, using tridecane as an internal standard. The extraction process was performed for 24 h in a shaker at 1000 rpm at 24 °C. The extract, filtered through 0.45 μm filters, was stored in vials at −20 °C before GC/FID analysis. Otherwise, 0.5 g of material was ground in liquid nitrogen and placed in vials for the GC/MS analysis.

### 4.3. Extraction of Phenolic Compounds

The fresh tissue samples (1 g) were ground in liquid nitrogen and extracted with ethanol (two steps) by alternating magnetic stirring and sonication, as already described in a previous study [48]. A successive liquid/liquid extraction with *n*-hexane (1:1, *v*/*v*) was applied to remove part of the chlorophylls, and the residual ethanol solutions were directly analyzed by HPLC.

### 4.4. GC/FID and GC/MS Analyses

The analyses were performed with the Gas Chromatograph Perkin-Elmer AutoSystem XL equipped with an automatic sampler for liquid sample injections and with the TotalChrom™ 6.2.0.0.0.:B27 chromatography software (Perkin Elmer Italia S.p.A., Milano (MI), Italy).

To obtain the separation of the enantiomeric monoterpenes, an Elite-Betacydex Betacyclodextrin capillary column (Perkin Elmer Italia S.p.A., Milano (MI), Italy), 30 m long and 0.25 mm in diameter, was used. GC analysis was carried out using hydrogen as a carrier gas at 2.0 mL·min^–1^ by a flame ionization detector at 250 °C and at injector temperature of 230 °C. The oven temperature programming started at 40 °C for 3 min and increased to 200 °C, at 1 °C·min^–1^, and the final temperature of 200 °C was maintained for 10 min. Volatile terpenes were identified by comparison of their retention times with those of standards under the same conditions. High-purity components were obtained from Sigma-Aldrich (Steinheim, Germany). Absolute amounts of terpenoids (total concentrations) were determined by comparison with the tridecane internal standard, and expressed as mg g^–1^ fresh weight (FW). Relative amount (proportion or percentage) of each monoterpene was expressed as a percentage of total monoterpenes (monoterpene profiles), while each sesquiterpene was calculated as a percentage of total monoterpenes plus sesquiterpenes (terpene profiles).

Headspace Gas Chromatography/Mass Spectrometry (HS-GC/MS) was then performed on a GC/MS system (Perkin Elmer Italia S.p.A., Milano (MI), Italy) composed of a GC AutoSystem XL coupled to a TurboMass mass spectrometer and combined with a TurboMatrix 40 automatic headspace sampler. The gas chromatograph was equipped with the column described above, operating with helium as a carrier gas at a constant flow of 3 mL min^−1^. The oven program was as follows: the initial temperature was 40 °C for 3 min, then a ramp of 1 °C min^−1^ brought the final temperature to 200 °C. The inlet temperature was 200 °C. The mass spectrometer was operating with an electron ionization of 70 eV, scanning the mass range from 35 to 350 *m*/*z*. Ion source temperature was 200 °C. The GC/MS control and data elaboration were performed by Perkin-Elmer Technologies TurboMass 5.4.2.1617 ver. Chemstation software. The mass spectrometer was calibrated using perfluorotributhylamine, as the calibration standard, with the Chemstation software.

Headspace gas chromatography (HS-GC) analyses were performed using the following conditions: He (carrier gas) at 3 mL min^−1^, pressurization time, 5 min, injection time set at 0.05 min to minimize peak broadening, thermostatting temperatures 80 °C, thermostatting times 30 min.

The MS spectral database (Wiley library) confirmed the elution sequence of the rosemary terpenes obtained by comparing the retention times of recorded peaks with those obtained by injecting pure compounds.

### 4.5. HPLC/DAD/ESI/MS Analyses

The analyses were carried out using a HP 1100 L liquid chromatograph equipped with a DAD detector, coupled to a HP 1100 MSD mass spectrometer with an API/electrospray interface (all from Agilent Technologies, Palo Alto, CA, USA). The analysis conditions were the same as those described in our previous study [28,49]. A Fusion RP18 column (150 × 2 mm i.d., 4 μm, Phenomenex, Torrance, CA, USA) was used and the mobile phases were (A) 0.1% formic acid/water and (B) CH_3_CN. The multi-step linear solvent gradient was: 0–15 min 15–25% B; 15–25 min, 25–35% B; 25–35 min 35–50% B; 35–40 min 50–100% B, with a final plateau of 8 min at 100% B, flow rate 0.2 mL·min^−1^ and oven temperature 26 °C, with an injection volume of 5 μL.

The quantitative evaluation of the main phenolic constituents was performed using three external standards: rosmarinic acid at 330 nm, genkwanin at 350 nm and carnosic acid at 284 nm. Genkwanin was used at 350 nm to quantify the flavonoids, while carnosic acid to determine the non-volatile diterpenoids. The calibration curve of rosmarinic acid (Sigma-Aldrich, Steinheim, Germany)) was in a linearity range between 0.1 and 1.8 μg, with R^2^ 0.9998, the calibration curve of carnosic acid (Sigma-Aldrich) was in the linearity range of 0.05–3.06 μg, with R^2^ 0.9998, and a five-point calibration curve of genkwanin (purity grade ≥ 95% by Extrasynthese) was obtained (range 0.01 to 0.85 μg), with an R^2^ 0.9999.

### 4.6. Microbial Strains and Antimicrobial Compounds

*Alternaria alternata* and *Pseudomonas viridiflava* strains belong to the microbial collection of the IPSP-CNR, Florence (Italy). The fungus *A. alternata* was grown on Potato Dextrose Agar (PDA, Oxoid Ltd., Basingstoke, UK) plates for 1 week at 25 °C. The bacterium *P. viridiflava* was grown on Nutrient Agar (Oxoid Ltd., UK) amended with 2.5 g/L of glucose (NGA) for 48 h at 27 °C. PDA and NGA media were also used to test the inhibition activity against the two pathogens of the main terpenic and phenolic compounds of rosemary leaf identified in this study. The selected compounds were: (+)-borneol, (−)-borneol, (+)-camphor, (−)-camphor, β-caryophillene, (+)-α-pinene, (−)-α-pinene, (+)-β-pinene, (−)-β-pinene, (−)-verbenone and rosmarinic acid. Copper sulphate was used as a positive control for both pathogens. The agar well-diffusion method was used to evaluate the antimicrobial activity of the different compounds [50].

#### 4.6.1. Antimicrobial Activity against *A. alternata*

PDA was prepared according to the manufacturer’s instructions, autoclaved at 121 °C for 15 min and poured into Petri dishes. After the medium solidification, two wells of 0.5 cm diameter were cut at the opposite side of the agar plates using a cork borer and 20 µL of the liquid or 20 µg of the solid compounds were placed into the wells. Twenty µL of sterile distilled water (SDW) was placed into the wells of control plates. Then, a 0.5 cm disk cut from the edge of the *A. alternata* colony was placed in the middle of each Petri dish. Three plates for each treatment were prepared. Plates were sealed with three layers of parafilm, incubated at 25 °C and monitored for two weeks. To evaluate the inhibition activity of the tested compounds against *A. alternata*, two diameters were measured for each fungal colony and data were compared to the diameter size of the control (SDW).

#### 4.6.2. Antimicrobial Activity against *P. viridiflava*

The 48 h-old culture of *P. viridiflava* was used to prepare a bacterial suspension in saline solution (0.8% NaCl). The bacterial concentration of the suspension was measured by a spectrophotometer and adjusted to an optical density of 0.1 at 530 nm, corresponding to 1 × 10^8^ cfu/mL, and 1.5 mL of the suspension was mixed with 15 mL of NGA medium poured into the Petri dishes. After the medium solidification, two wells of 0.5 cm diameter were cut at the opposite side of the agar plates using a cork borer, and then 20 µL of the liquid or 20 µg of the solid compounds were placed into the wells. Plates with 20 µL of SDW in the wells were used as controls of bacterial growth. Three plates for each treatment were prepared. Plates were sealed with three layers of parafilm and then incubated at 27 °C for 48 h. The antibacterial activity of the tested compounds was evaluated measuring the size of the growth inhibition halo surrounding the wells.

#### 4.6.3. Determination of the Minimum Inhibitory Concentration (MIC)

*A. alternata*: The 96-well microplate dilution method reported by Hassan and Cutter [51] was used for (+)-α-pinene, (−)-β-pinene, verbenone, rosmarinic acid and copper sulphate, while for borneol, and the two camphor enantiomers that were not soluble in the liquid growth medium, the protocol described above (Section 4.6.1) was used to determine the MIC. For the microdilution method, the fungus was grown on PDA plates for one week, then conidia were collected by scraping the agar surface and suspended in potato dextrose broth (PDB). Conidia were enumerated by a hemocytometer and concentration was adjusted to 1 × 10^6^ conidia/mL. The antimicrobial compounds were dissolved in methanol to obtain a 10% solution. This solution was serially diluted 1:1 in PDB until the concentration of 0.039% [51]. One hundred µL of each dilution were placed in the microplate wells, then 100 µL of the conidial suspension was added to each well. Each dilution was tested in triplicate. The positive control was represented by 100 µL of the conidial suspension plus 100 µL of PDB, while the negative control consisted of 200 µL of sterile PDB. A control to test methanol inhibition activity was also carried out. Plates were incubated at 25 °C for 48 h and fungal growth was determined both by visual reading and by a spectrophotometer at 450 nm. The MIC value for the two camphor enantiomers and borneol was considered as the highest dilution of each compound that determines a significant reduction of the growth of *A. alternaria* colony with respect to the control (SDW).

*P. viridiflava*: The 96-well microplate dilution method was used for all compounds. The bacterium was grown for 48 h in plates containing NGA medium, then the bacterial suspension was prepared in nutrient broth amended with 0.25% glucose (NGB) following the procedure described in Section 4.6.2. On the basis of the results obtained from the test of in vitro antimicrobial activity, only the compounds that were active against *P. viridiflava* (i.e., (+)-α-pinene, verbenone and rosmarinic acid) were assayed for the MIC determination. Only the enantiomer (+)-α-pinene was tested, since inhibition activity had resulted not different for the two forms of this compound. The dilutions of the different compounds and next steps of the procedure were the same of that previously reported for *A. alternata*, using NGB as a growth medium. The positive control was represented by 100 µL of the bacterial suspension plus 100 µL of NGB, while the negative control consisted of 200 µL of sterile NGB. A control to test methanol inhibition activity was also carried out. Plates were incubated at 27 °C for 24 h, then the bacterial growth was first recorded by a visual reading of the plates. Then, 30 µL of a resazurin solution (0.015%) was added to each well and plates were incubated for 3 h at 27 °C. The color of the suspensions contained in each well was recorded.

### 4.7. Statistical Analyses

Data did not meet the normality assumption of ANOVA even after transformation (Kolmogorov–Smirnov one-sample test), and therefore were analyzed by the non-parametric Kruskal–Wallis ANOVA followed by the Mann–Whitney U-test for multiple comparisons using SYSTAT 12.0 software (Systat Software Inc., Richmond, CA, USA). Differences were accepted when significant at the 5% level.

## 5. Conclusions

Our results showed significant changes in total concentrations and relative contents of terpenes and their enantiomeric distribution within both different tissue sources and tissue ages.

The highest concentration was detected in young leaves, while the lowest absolute amount in xylem tissue samples. The young cortex contained appreciable contents that can affect the oil composition and increase yield.

Among the phenolic compounds, the contents of only four minor flavonoids showed variations in foliar tissue samples collected at different ages.

Microbial test results showed that several terpenes and rosmarinic acid inhibited the growth of two polyphagous pathogens of rosemary, the fungus *A. alternata* and the bacterium *P. viridiflava*. Knowledge of chemical composition variations due to source and age of tissues is important for a more targeted harvesting of the rosemary plant, which can help to produce higher-quality essential oils and richer phenolic extracts for agro-food, pharmaceutical and cosmetics applications. In addition, results on antimicrobial activity of selected terpenes and rosmarinic acid suggest the use of these natural compounds as biochemical markers in breeding programs for the selection of less susceptible rosemary chemotypes, and to produce eco-friendly chemical alternatives to synthetic pesticides for improving human and environmental health.

## Figures and Tables

**Table 1:** Average amounts (mg/g FW) of phenolic compounds in young leaves (LY) and mature foliar tissue samples collected from the upper part of the branch (LMu). FW, fresh weight; SE, standard error.

	LY		LMu		
	mg/g FW	SE	mg/g FW	SE	
Isoorientin (luteolin 6-*C*-glu)	0.63 ^b^	0.05	0.42 ^a^	0.01	*
Homoplantaginin (hispidulin 7-*O*-glu)	0.62 ^b^	0.04	0.35 ^a^	0.05	*
Rosmarinic acid	5.24	0.79	3.32	0.67	NS
Isoscutellarein 7-*O*-glu	0.6	0.05	0.51	0.05	NS
Flavonoid 1 **^$^**	0.48	0.02	0.51	0.04	NS
Cirsimaritin	0.67 ^a^	0.04	0.98 ^b^	0.1	*
Flavonoid 2 **^$^**	0.54	0.03	0.49	0.03	NS
Genkwanin	0.46 ^a^	0.02	0.92 ^b^	0.13	*
4′-methoxytectochrysin	0.62	0.03	0.74	0.08	NS
Carnosol	12.87	3.78	8.27	4.01	NS
Carnosic acid	28.55	4.68	17.41	1.51	NS
Methyl carnosic acid	2.07	0.24	1.91	0.43	NS
Total flavonoids	4.62	0.24	4.92	0.34	NS
Total terpenoids	43.49	8.2	27.59	4.05	NS
Total phenols	53.35	8.82	35.83	4.82	NS

* *p* < 0.05; NS, not significant; glu, glucose. ^$^ These compounds were tentatively identified as flavonoids according to their UV-Vis and mass spectra. “a” and “b” letters indicate significant differences (by the Mann–Whitney U-test, *p* < 0.05) in the content of each single phenol between LY and LMu samples.

**Table 2:** Minimum inhibitory concentrations (MIC) of (+)-α-pinene, (−)-β-pinene, (−)-verbenone, rosmarinic acid and copper sulphate against *A. alternata* and *P. viridiflava* determined by the microdilution method and expressed as percentage.

	*A. alternata*		*P. viridiflava*	
	V.R.	S.R.	V.R.	Resazurin
(+)-α-pinene	0.625	0.625	1.25	1.25
(−)-β-pinene	0.313	0.313	n.i.	n.i.
(−)-verbenone	0.625	0.625	1.25	1.25
rosmarinic acid	n.d.	1.25	1.25	1.25
copper sulphate	0.078	0.078	0.625	0.313

V.R.—visual reading; S.R.—spectrophotometric reading; n.i.—not investigated; n.d.—not detectable.

**Table 3:** Minimum inhibitory concentrations (MIC) of borneol, (+)-camphor and (−)-camphor against *A. alternata* determined by the agar well-diffusion method. Data represent the means of 4 replicates of the diameter size (cm) of *A. alternata* colonies.

	Borneol	(+)-Camphor	(−)-Camphor
SDW	6.4 ^d^	6.4 ^c^	6.4 ^c^
5%	3.8 ^a^	4.2 ^a^	3.7 ^a^
2.5%	4.5 ^b^	4.5 ^ab^	4.9 ^b^
1.25%	5.2 ^c^	4.9 ^b^	5.1 ^b^
0.625%	6.2 ^d^	5.0 ^b^	5.1 ^b^
0.313%	6.2 ^d^	6.2 ^c^	6.6 ^c^

Different letters indicate significant differences (by the Mann–Whitney U-test, *p* < 0.05) among the terpenes. In bold are shown the MIC values for each compound.

## Data Availability

The data presented in this study are available on request from the corresponding author.

## Supplementary Materials

Table S1: List of terpenes and the main phenolic compounds identified in rosemary extracts. Rt, retention times; MW, molecular weight. Table S2: Statistical results of the Kruskal–Wallis ANOVA test examining variations in the relative content of terpenes in relation to source and age of the tissue: N = 63; degrees of freedom = 6; *χ*^2^ value; *p*-value where *** *p* < 0.001. L, leaf; B, cortex; X, xylem tissue; Y, young; M, mature. Samples collected from the upper part of the branch are indicated with “u” and at the base of the branch with “b”. Table S3: Determination of MIC of (+)-α-pinene, (−)-β-pinene, (−)-verbenone, rosmarinic acid and copper sulphate against *A. alternata* by the microdilution method. Data represent the spectrophotometer absorbance values measured at 450 nm. Values in bold indicate the MIC values. Control value is 0.188 (average value of eight measurements) and is represented by *A. alternata* grown without antimicrobials. At concentration of 0.313% of rosmarinic acid, *A. alternata* releases a dark pigment in the growth medium. Figure S1: Response of the fungus *A. alternaria* to rosmarinic acid. Left: plate with wells containing SDW; right: plate with wells containing rosmarinic acid. Figure S2: Response of the bacterium *P. viridiflava* to rosmarinic acid. Left: plate with wells containing SDW, the bacterium covers the agar surface as a pale-yellow thin layer. Right: plate with wells containing rosmarinic acid, the pale-yellow layer is hidden by the dark pigment, and around the wells, no bacterial growth was observed. Figure S3: Determination of MIC for (+)-α-pinene (columns 1–3), (−)-verbenone (columns 4–6) and rosmarinic acid (columns 7–9) against *P. viridiflava*. After 24 h of incubation, 30 µL of a resazurin solution was added to each well. The blue color indicates no bacteria multiplication (column 12, negative control), while the pink/red color indicates that the bacterial growth was not inhibited (column 10, positive control). Column 11 represents the control for methanol since all dilutions of the different compounds were made in this solvent.

## Sample Availability

Samples of the compounds are not available from the authors.
