# Supplementary material for: Within-Plant Variation in Rosmarinus officinalis L. Terpenes and Phenols and Their Antimicrobial Activity against the Rosemary Phytopathogens Alternaria alternata and Pseudomonas viridiflava
Source: Molecules. 2021 Jun 5;26(11):3425. doi: 10.3390/molecules26113425 (PMC8201224; doi:10.3390/molecules26113425)
Supplement: Supplementary file 1 [file molecules-26-03425-s001.zip › molecules-1214706-supplementary.pdf]

Supplementary Materials

Table S1. List of terpenes and the main phenolic compounds identified in rosemary extracts. Rt, retention times; MW, molecular weight.

Compounds	Chemical structure	Rt (min)	MW
**Terpenes**			
(-)-α-Pinene	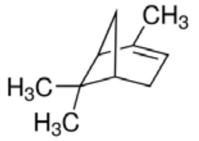	12.1	136.23
(+)-α-Pinene	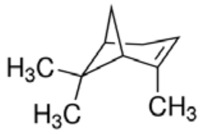	12.6	136.23
Camphene	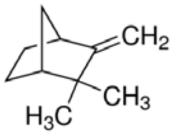	14.0	136.23
Sabinene	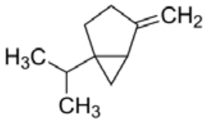	14.2	136.23
Myrcene	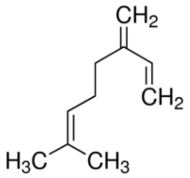	16.2	136.23
(+)-β-Pinene	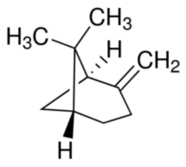	16.5	136.23
(-)-β-Pinene	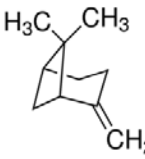	16.6	136.23
(-)-Limonene	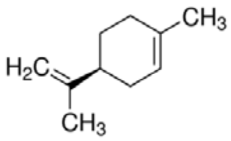	19.3	136.23
(+)-Limonene	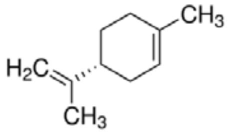	19.4	136.23
p-Cymene	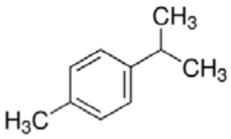	20.3	134.22

1,8-Cineole	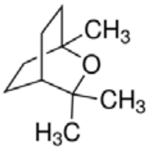	24.1	154.25
(-)-Linalool	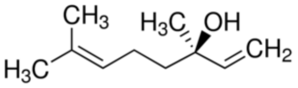	35.2	154.25
Camphor	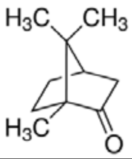	39.8	152.23
Terpinen-4-ol	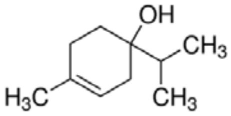	41.7	154.25
Borneol	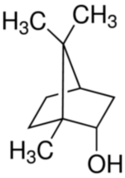	46.5	154.25
(-)-Bornylacetate	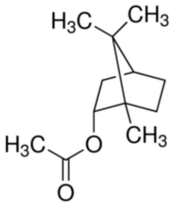	46.7	196.29
(-)-Verbenone	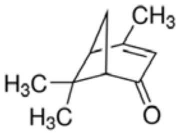	47.9	150.22
Geraniol	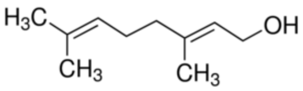	49.3	154.25
β-Caryophyllene	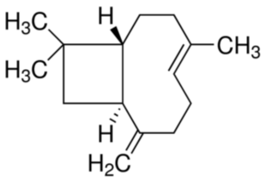	54.2	204.35
Geranyl acetate	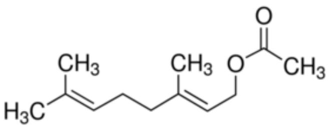	55.7	196.29
α-Humulene	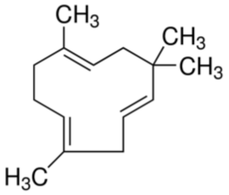	57.4	204.35
Eugenol	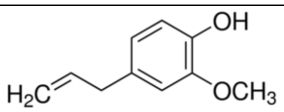	59.4	164.20

Thymol	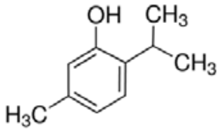	67.1	150.22
Carvacrol	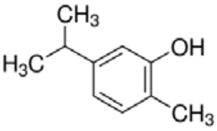	71.5	150.22
Caryophyllene oxide	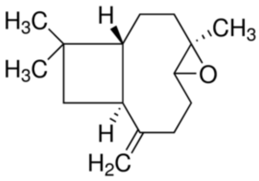	73.1	220.35
**Phenolic compounds**			
Caffeic acid	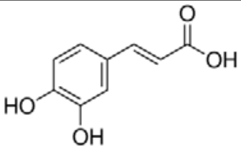	8.2	180.16
Isoorientin (luteolin 6-*C*-glucoside)	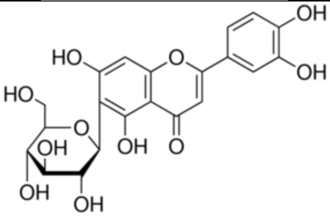	16.7	448.38
Hesperidin	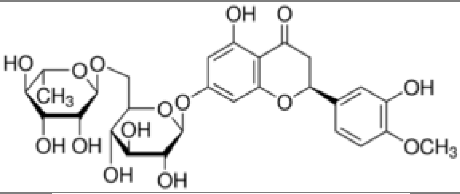	18.5	610.56
Homoplantaginin (hispidulin 7-*O*-glucoside)	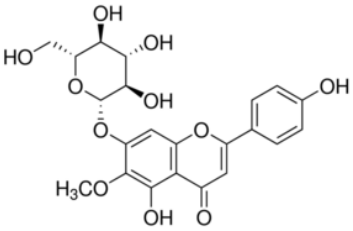	20.3	462.40
Rosmarinic acid	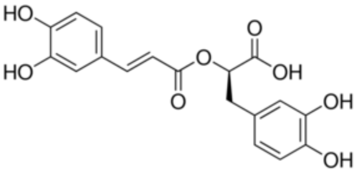	21.8	360.31
Luteolin 7-*O*-glucuronide	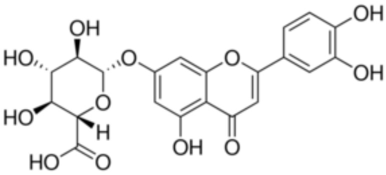	23.0	462.36
Isoscutellarein 7-*O*-glucoside	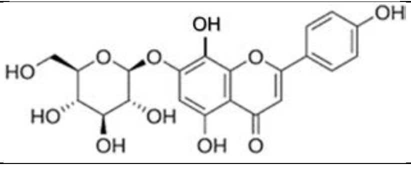	25.8	448.4

Cirsimaritin	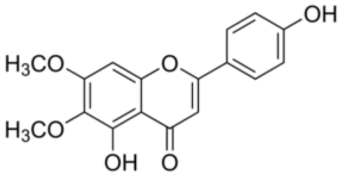	38.1	314.29
Genkwanin	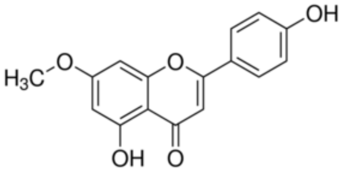	41.8	284.26
4′-Methoxytectochrysin	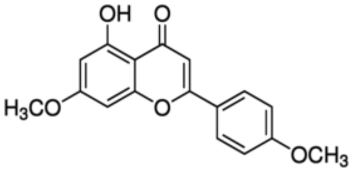	44.0	298.29
Carnosol	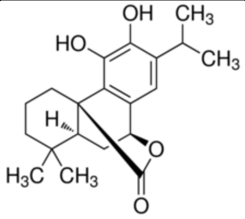	44.9	330.42
Carnosic acid	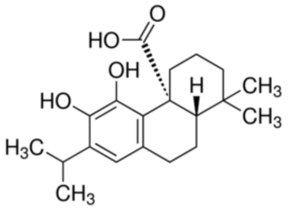	46.0	332.43

Table S2. Statistical results of the Kruskal–Wallis ANOVA test examining variations in the relative content of terpenes in relation to source and age of the tissue: N=63; degrees of freedom=6; χ^2 value; *p*-value where *** p<0.001. L, leaf; B, cortex; X, xylem tissue; Y, young; M, mature. Samples collected from the upper part of the branch are indicated with "u" and at the base of the branch with "b".

Terpene	$\chi^2(p)$	LY	LMu	LMb	BY	BM	XY	XM
(-)-α-pinene	27.2***	c	c	c	d	cd	b	a
(+)-α-pinene	32.9***	c	c	c	c	c	b	a
(-)-camphene	34.7***	b	b	b	c	c	b	a
sabinene	40.2***	b	c	c	b	b	a	a
myrcene	40.8***	b	c	c	b	a	a	a
(+)/(-)-β-pinene	21.9***	b	a	a	b	c	d	d
(-)-limonene	49.6***	b	c	c	b	b	a	a
(+)-limonene	52.4***	b	c	c	b	b	a	a
p-cymene	40.6***	c	c	c	c	b	a	a
1,8-cineole	26.8***	c	c	c	b	b	a	a
(-)-linalool	55.5***	b	c	c	a	a	a	a
camphor	39.7***	b	b	b	b	b	a	a
terpinen-4-ol	40.1***	c	c	c	b	b	a	a
borneol	27.4***	c	bc	bc	c	bc	b	a
(-)-bornylacetate	33.4***	c	b	b	b	a	a	a
(-)-verbenone	49.9***	b	c	c	b	a	a	a

β-caryophyllene	42.8***	d	c	c	b	b	b	a
α-humulene	38.6***	d	c	c	b	a	a	a
caryophyllene oxide	34.6***	bc	b	b	c	c	a	a

Different letters indicated significant difference (by the Mann-Whitney U-test) in the content of each terpene among the different samples.

Table S3. Determination of MIC of (+)-α-pinene, (-)-β-pinene, (-)-verbenone, rosmarinic acid and copper sulphate against *A. alternata* by microdilution method. Data represent the spectrophotometer absorbance values measured at 450 nm. Values in bold indicate the MIC values. Control value is 0.188 (average value of eight measurements) and is represented by *A. alternata* grown without antimicrobials. At concentration of 0.313% of rosmarinic acid, *A. alternata* releases dark pigment in the growth medium.

Concentration (v/v or w/v)	(+)-α-pinene			(-)-β-pinene			(-)-verbenone			rosmarinic acid			copper sulphate		
5%	0.002	0.004	0.002	0.001	0.002	0.002	0.001	0.005	0.001	0.001	0.003	0.002	0.001	0.001	0.004
2.5%	0.000	0.001	0.003	0.003	0.000	0.002	0.001	0.002	0.003	0.003	0.003	0.001	0.003	0.002	0.001
1.25%	0.002	0.002	0.004	0.002	0.001	0.001	0.006	0.002	0.001	**0.002**	**0.001**	**0.002**	0.001	0.001	0.002
0.625%	**0.002**	**0.003**	**0.000**	0.002	0.003	0.006	**0.002**	**0.001**	**0.003**	0.153	0.164	0.200	0.003	0.002	0.002
0.313%	0.118	0.078	0.064	**0.005**	**0.003**	**0.001**	0.065	0.081	0.097	3.060	3.118	2.996	0.002	0.001	0.002
0.156%	0.235	0.163	0.147	0.091	0.079	0.076	0.115	0.120	0.146	0.190	0.104	0.114	0.005	0.003	0.001
0.078%	0.223	0.187	0.184	0.133	0.125	0.137	0.172	0.147	0.175	0.039	0.031	0.683	**0.003**	**0.001**	**0.001**
0.039%	0.257	0.257	0.212	0.182	0.200	0.189	0.265	0.250	0.244	0.188	0.253	0.234	0.017	0.011	0.028

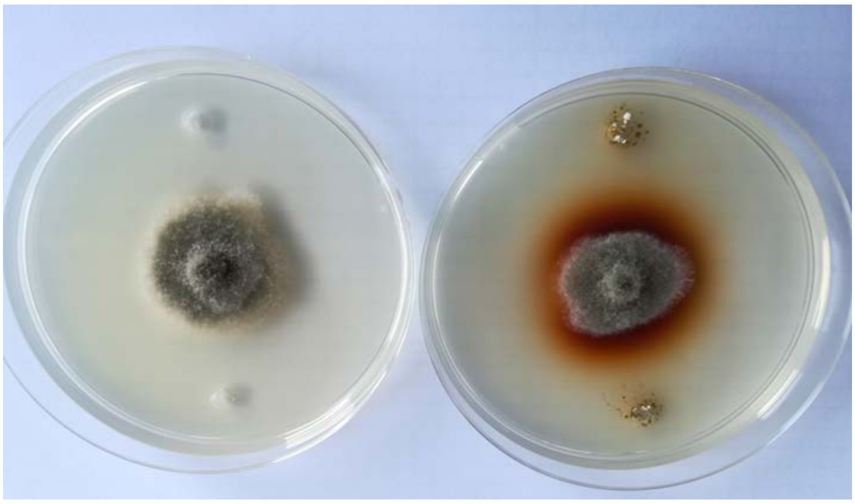

Figure S1. Response of the fungus *A. alternaria* to rosmarinic acid. Left: plate with wells containing SDW; right: plate with wells containing rosmarinic acid.

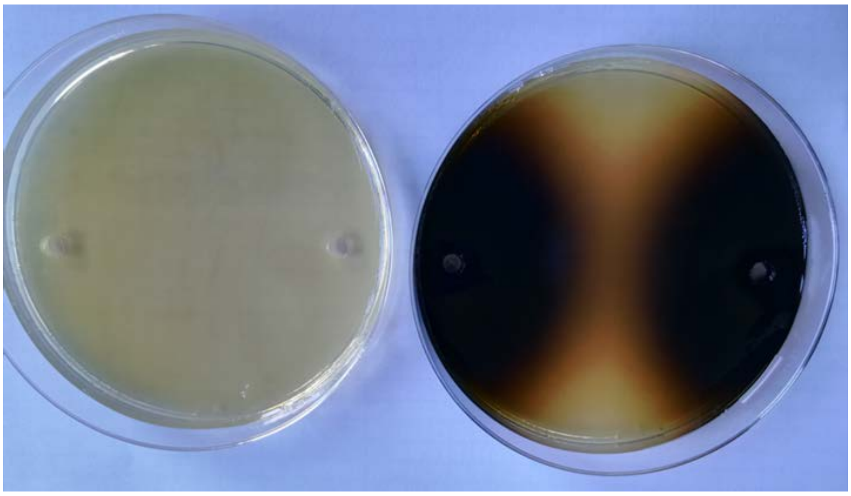

Figure S2. Response of the bacterium *P. viridiflava* to rosmarinic acid. Left: plate with wells containing SDW; the bacterium covers the agar surface as a pale yellow thin layer. Right: plate with wells containing rosmarinic acid; the pale yellow layer is hidden by the dark pigment, and around the wells no bacterial growth was observed.

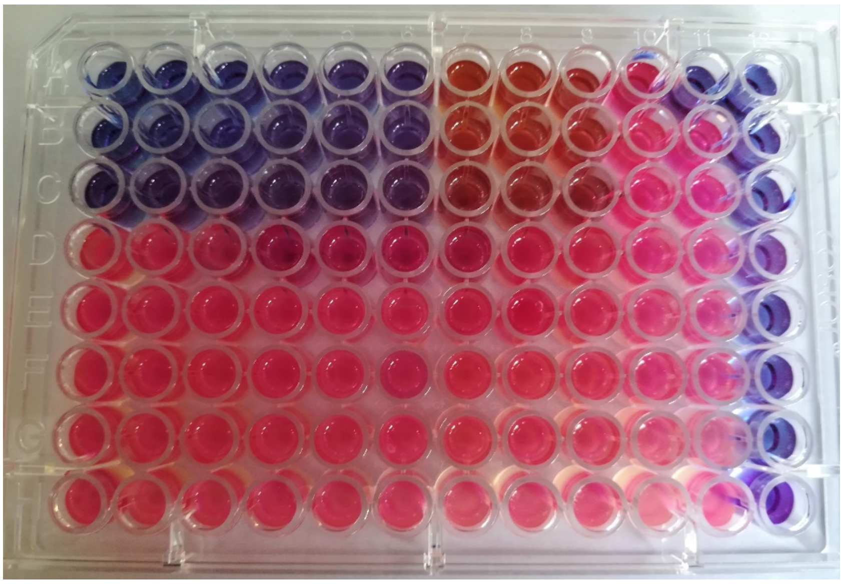

Figure S3. Determination of MIC for (+)-α-pinene (columns 1-3), (-)-verbenone (columns 4-6) and rosmarinic acid (columns 7-9) against *P. viridiflava*. After 24 h of incubation, 30 µL of a resazurin solution was added to each well. The blue colour indicates no bacteria multiplication (column 12 negative control), while the pink/red colour indicates that the bacterial growth was not inhibited (column 10 positive control). The column 11 represents the control for methanol since all dilutions of the different compounds were made in this solvent.
